# Group meetings of caretakers of patients with schizophrenia and bipolar mood disorders

**DOI:** 10.4103/0019-5545.55939

**Published:** 2005

**Authors:** T.M. Ismail Shihabuddeen, P.S. Gopinath

**Affiliations:** * Psychiatric Rehabilitation Practitioner, Department of Psychiatry, Father Muller Medical College, Mangalore, Karnataka; ** Emeritus Professor, Department of Psychiatry, Father Muller Medical College, Mangalore, Karnataka

**Keywords:** Caretakers'group meeting, schizophrenia, bipolar mood disorders

## Abstract

**Background:**

In India, there is a paucity of trained professionals to execute psychosocial interventions. Families are thus assigned the role of primary caretakers of individuals with chronic mental illness.

**Aim:**

To assess the perceived benefits and difficulties of group meetings among caregivers of persons with schizophrenia and bipolar mood disorders, and to evaluate the utilization pattern of general hospital psychiatric unit (GHPU) services by caregivers who regularly attend such group meetings.

**Methods:**

Persons with schizophrenia and those with bipolar mood disorders with associated psychosocial problems and on maintenance medication were identified at the Psychiatric Rehabilitation Unit of the Department of Psychiatry, Father Muller Medical College. Significant caregivers of the identified patients were informed about the group meeting. Group meetings were conducted for about 45 minutes once a month for caregivers of patients with schizophrenia and bipolar mood disorders. Data regarding the psychosocial aspects of caregivers were collected before entry to the meeting and after 17 months of their participation. Participants who attended the meetings irregularly were excluded from the study.

**Results:**

The group meetings led to effective monitoring of the functioning of individuals, a reduction in the subjective family burden and family distress, a better support system with adequate coping skills and good compliance with treatment programmes.

**Conclusion:**

Conducting regular group meetings for a homogeneous population at a GHPU is feasible and beneficial.

## INTRODUCTION

In India, families represent the key resource persons in the care of patients with chronic mental illness. Families are assigned the role of primary caretakers for two reasons. First, there is a paucity of trained professionals required to execute psychosocial interventions and second, most Indian families would like to be meaningfully involved in all aspects of care of their ill relative.^1^ Social stigma and misconception often prevent the family from seeking proper assistance.^2^ The different ways in which families view, interpret and cope with psychiatric disorders, the role of religion and traditional treatments as well as how culture might shape the attitudes of service providers in psychosocial rehabilitation should be borne in mind. The families want understanding of the symptoms, specific suggestions for coping with clients' behaviour and relating to people with similar experiences.^3^ Significant family members need persuasion to erase ignorance and to get educated to help themselves by utilizing available facilities. Families need to be convinced that, in addition to clinical treatment, rehabilitation programmes bring the client into the mainstream of society.^4^ Family interventions have assumed greater importance as a result of the shift of clients from the hospital to the community. The process of care and rehabilitation often takes a long time and places heavy responsibilities and financial constraints on the family. Caregivers need coping skills, social support and active engagement in the educational process. They are encouraged by involving them as collaborators at all stages of treatment. As much as possible, empirically validated treatment principles drawn from the clinical psychiatric literature are used in psychoeducation, carefully modified to the level of comprehension of laypersons.^5^

Family psychoeducational interventions can help family members cope with various distressing symptoms.^6^ Studies show that family psychoeducation programmes have significant benefits in areas other than symptom and relapse management for the family member with a chronic psychiatric disorder.^4^ When psychoeducation and skills training are conducted in a multiple family group format, family burden is lowered and relatives' sense of self-efficacy regarding the ill family member improves. Participating in a multiple family group meeting can reduce the stress, isolation and stigma experienced by family members; the burden of care no longer seems unique, and families can exchange helpful suggestions and coping techniques with each other. Evaluation of the clinical effect of multiple family group meetings suggests that the format may be at least as effective as single family psychoeducation in reducing relapse.^5^ Family intervention increases the level of the patient's social competence, decreases the subjective burden on relatives, changes the communication pattern and the overall interaction within the family.^7^

Much has been discussed about the need for development in the area of psychosocial rehabilitation. Though there have been sporadic efforts in this area in India, there is an urgent need to discuss the outcome of these innovative approaches on a common platform, to evaluate the feasibility and benefits of such programmes. The role that families/caregivers play in managing an individual with a psychiatric disorder has gained increasing attention in the past few years. In the Indian context, families are the primary care personnel who need to provide sustained, long-term care of reasonable quality to the majority of people disabled by mental illness. Therefore, any attempt to assist family members is likely to enhance the care they can provide to the individual with disability who is the focus of the rehabilitation effort.^1^

The present study was carried out to assess the perceived benefits of group meetings of caregivers of those with schizophrenia and bipolar mood disorder, and evaluate the utilization pattern of general hospital psychiatric unit (GHPU) services by those who regularly participate in group meetings.

## METHODS

Caregivers of those with schizophrenia and bipolar mood disorders with associated psychosocial problems and on maintenance medication were identified for group meetings at the Psychiatric Rehabilitation (PSR) Unit of the Department of Psychiatry, Father Muller Medical College. The psychosocial problems noticed during the baseline assessment were mainly in the areas of subjective family burden,^8^ compliance behaviour,^9^ knowledge regarding the illness and treatment, family distress, caretaker's attitude towards the client and the social support system.^10^ After the baseline assessment, information regarding the purpose of the group meeting and the timings were announced. The inclusion criterion was that the participant must be the primary caregiver who stays with the client and regularly attends the OPD. Caregivers of persons with any co-morbid conditions were excluded from the study. Group meetings were conducted for caregivers once a month for both the schizophrenia group and the bipolar mood disorder group for 45–60 minutes. Each group meeting followed a more or less structured format (Box 1). The initial stages of the group meeting were followed by a psychoeducational approach which later led to follow-up of a ‘combined group process and psychoeducational approach’,^11^ and a problem-solving approach. Data collected from the caregivers after 17 months of group interventions were studied to understand the effectiveness of the meetings. The follow-up dates in the OPD for persons with schizophrenia and those with bipolar mood disorder are given on the first and third Thursday of every month, respectively.

**Box 1:** Format of group meetings of caregivers

**Initial phase:** All the participants registered their names before the start of the meeting. Refreshments were provided before the self-introductory session to promote feelings of oneness and participation. The leader of the meeting and the topic were selected by the group members. **Middle phase:** The leader read out the review of the previous meeting. A group discussion on the selected topic was prompted by a therapist followed by a doubtclearing session. The middle phase ended with a discussion of the clarified doubts. **Termination phase:** A summary of the group meeting was presented by the group leader. The group concluded the meeting after announcement of the date and time of the next meeting.

There were 46 participants (23 in each group). Their ages ranged from 18 to 59 years, with a mean of 28.565±8.63 years in the schizophrenia group and 33.04±13.53 years in the bipolar mood disorder group; the p value was 0.18, which was not significant (Table 1). The majority of participants were in the age group of 20–29 years.

**Table 1:** Sociodemographic profile of the participants (*n*=46)

Variable	Group A	Group B
Age (mean±SD) (years)	28.565±8.63	33.040±13.53
Gender				
Male	13	11
Female	10	12
Duration of illness				
1 year or less	1	8
Up to 5 years	13	8
Up to 10 years	7	4
Up to 20 years	2	2
More than 20 years	—	1
Domicile				
Urban	4	1
Semi-urban	5	15
Rural	14	7
Socioeconomic status				
High	1	3
Middle	13	15
Low	9	5

Group A: caregivers of persons with schizophrenia; Group B: caregivers of persons with bipolar mood disorder

## RESULTS

**Fig. 1:**
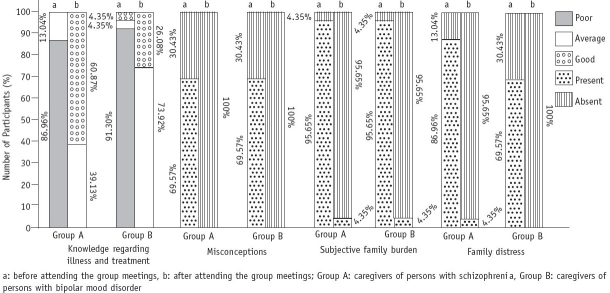
Comparison of the effectiveness of group meetings among caregivers of patients with schizophrenia and bipolar mood disorder

There was significant improvement in knowledge regarding the illness and treatment after attending the group meetings. In both the groups, 95.65% of the participants had a subjective family burden before availing of the group interventions and only 1 participant (4.35%) in each group felt burdened after the interventions. There was significant improvement in the area of family distress (Fig. 1).

Among the participants, 40 (86.96%) had a weak support system; regular attendance of the group meetings strengthened the support system of 45 participants (97.83%). Thirty-nine caregivers (69.57%) could learn adequate coping skills after the group interventions. In the schizophrenia group, 7 patients (30.44%) were poorly compliant and 16 (69.57%) were supervised in medication intake, before their caregivers started attending the group meetings, whereas 1 (4.35%) was supervised and 22 (95.65%) had good compliance with the prescribed treatment programme after the caregivers started participating regularly in the group meetings. In the bipolar mood disorder group, 16 patients (69.57%) were poorly compliant and 7 (30.44%) were supervised in medication intake before the caregivers started attending the group meetings, whereas 12 (52.18%) had good compliance with the prescribed treatment regimen after the group interventions. All the caregivers from the schizophrenia group and 95.65% from the bipolar mood disorder group had a positive attitude towards their ward after attending the group meetings regularly (Table 2).

**Table 2:** Changes in psychosocial aspects after the group meetings among caretakers of patients with schizophrenia and bipolar mood disorder

	Schizophrenia (n [f])		Bipolar mood disorder (n [f])	
		
Psychosocial aspect	a	b	a	b
Family support system of the client				
Weak	19 (82)	1 (4.35)	21 (91.3)	0
Good	4 (17.4)	22 (95.65)	2 (8.7)	23 (100)
Coping skills of caregiver				
Inadequate	22 (95.65)	1 (4.36)	23 (100)	6 (26.06)
Adequate	1 (4.35)	22 (95.65)	0	17 (73.9)
Treatment compliance				
Poor	7 (30.43)	0	16 (69.57)	0
Supervised	16 (69.57)	1 (4.35)	7 (30.43)	11 (47.38)
Good	0	22 (95.65)	0	12 (52.18)
Attitude of the caregiver towards the client		
Negative	13 (56.12)	0	14 (60.87)	1 (4.35)
Positive	10 (43.48)	23 (100)	9 (39.13)	22 (95.63)

a: before the group meeting; b: after the group meeting

## DISCUSSION

Several modes of family interventions have been designed and empirically validated for caregivers of persons with schizophrenia and bipolar mood disorders to equip them with coping skills and thereby change the emotional climate of the family and reduce the incidence of relapse. However, hardly any literature is available on the efficacy of group meetings in the Indian setting. Since the 1970s, a number of different approaches have come up for working with families.

The findings of the present study reveal that group interventions are beneficial for caregivers of persons with schizophrenia as well as of those with bipolar mood disorder. Both the groups showed significant improvement in the areas of subjective family burden, family distress, compliance behaviour and coping skills. These results are supported by the findings that multiple family group meetings on schizophrenia resulted in a reduction of family distress.^12^ Psychoeducational groups for caregivers of patients with bipolar mood disorder improve their knowledge regarding the illness, and this reduces their distress or subjective burden.^13^ From these results it can be concluded that participating in multiple family groups can reduce the stress, isolation and stigma experienced by caregivers; the burden of care no longer seems unique, and families can exchange helpful suggestions and coping techniques with each other. We could also strengthen the support system of the clients after 17 of these meetings. When families are well informed about the mental illness, they can become allies in treatment, control stress and participate in problem-solving.^14^

Introduction of the group intervention did not interfere with the regular OPD functioning. We subdivided both the groups into two, based on the language of communication for better group interaction and equal participation. Limitations of the study include the small sample size and lack of use of standardized research tools for the assessment of parameters. It is recommended that future research must have a comparative design and be done using standard tools before recruitment of the group intervention and after interventions.

## CONCLUSION

Conducting regular group meetings at a GHPU is feasible for care providers and beneficial for those with schizophrenia and bipolar mood disorder.
